# Antihistamines, phenothiazine-based antipsychotics, and tricyclic antidepressants potently activate pharmacologically relevant human carbonic anhydrase isoforms II and VII

**DOI:** 10.1080/14756366.2023.2188147

**Published:** 2023-03-13

**Authors:** Francesco Fiorentino, Alessio Nocentini, Dante Rotili, Claudiu T. Supuran, Antonello Mai

**Affiliations:** a Department of Drug Chemistry and Technologies, Sapienza University of Rome, Rome, Italy; b Department of NEUROFARBA, Section of Pharmaceutical and Nutraceutical Sciences, Polo Scientifico, University of Florence, Firenze, Italy; c Pasteur Institute, Cenci-Bolognetti Foundation, Sapienza University of Rome, Rome, Italy

**Keywords:** Carbonic anhydrase activators, antihistamines, antipsychotics, antidepressants, neurodegenerative diseases

## Abstract

Carbonic anhydrases (CAs) are important regulators of pH homeostasis and participate in many physiological and pathological processes. CA activators (CAAs) are becoming increasingly important in the biomedical field since enhancing CA activity may have beneficial effects at neurological level. Here, we investigate selected antihistamines, phenothiazine-based antipsychotics, and tricyclic antidepressants (TCAs) as potential activators of human CAs I, II, IV, and VII. Our findings indicate that these compounds are more effective at activating hCA II and VII compared to hCA I and IV. Overall, hCA VII was the most efficiently activated isoform, particularly by phenothiazines and TCAs. This is especially relevant since hCA VII is the most abundant isoform in the central nervous system (CNS) and is implicated in neuronal signalling and bicarbonate balance regulation. This study offers additional insights into the pharmacological profiles of clinically employed drugs and sets the ground for the development of novel optimised CAAs.

## Introduction

Organisms in all kingdoms of life function in a water-based medium and rely on a plethora of biochemical transformations of the carbon element. In this context, the reversible hydration of carbon dioxide (CO_2_) into water-soluble bicarbonate (HCO_3_^-^) and proton ions, chemically represented by Equation (1), is of utmost importance.^1–3^

(1) $${\mathrm{CO}}_{2}+H_{2}O\;⇌\;{\mathrm{HCO}}_{3}^{-}+H^{+}$$

Nonetheless, at typical intracellular CO_2_ concentrations, this reaction is incredibly slow as CO_2_ is weakly soluble in water, with k_cat_ values for the uncatalysed reaction of 0.15 s^−1^ and 50 s^−1^ for the hydration and dehydration reactions, respectively.^4^^,^^5^ Hence, the metalloenzyme superfamily of carbonic anhydrases (CAs), which catalyse the reversible hydration of CO_2_, has a central role in regulating cell and organism homeostasis.^6^^,^^7^ Given the nature of the catalysed reactions, CA activity is pivotal for controlling pH and maintaining the acid-base balance, thereby being involved in the metabolism regulation under both physiological and pathological conditions.^8–10^

The exceptional physiological importance of these enzymes is testified by the existence of eight different genetic families (α-, β-, γ-, δ-, ζ-, η-, θ-, and ι-CAs) and the multitude of isoforms present in each of them, which represents one of the most dramatic instances of convergent evolution in biology.^4^^,^^11–14^ In vertebrates, at least 16 CA isoforms are known, all belonging to the α-CA family, and 15 different CAs are expressed in humans, 12 of which are catalytically active.^2^ The fundamental differences between the different α-CA isoforms are mostly linked to their secondary and tertiary structures which influence their chemical and physical stability. The catalytic site, which is shared by all family members, comprises a Zn^2+^ ion coordinated by three histidine residues and a H_2_O/OH^-^ molecule.^2^

The catalytically active human CAs (hCAs) are distinct based on their subcellular localisation: hCA I-III, VII, and XIII are cytosolic;^15^^,^^16^ hCA IV is membrane-anchored *via* a glycosylphosphatiydyl-inositol and faces the extracellular space;^17^^,^^18^ hCA VA and VB are mitochondrial;^19^ hCA VI is secreted (in saliva and tears);^20^^,^^21^ hCA IX, XII, and XIV are transmembrane enzymes.^22^^,^^23^ Over the years, numerous studies have identified hCAs as drug targets and many CA inhibitors are of pharmacological interest for the treatment of epilepsy, oedema, glaucoma, obesity, and cancer.^6^^,^^24–27^hCAs are widely distributed in human tissues, and many of them are highly expressed in the central nervous system (CNS) and choroid plexus.^7^^,^^8^^,^^16^ Regarding the cytosolic CAs, the only one absent in the CNS is hCA XIII. The other cytosolic isoforms are all present in the CNS, with hCA VII, localised in the cortex, thalamus, and hippocampus, being the most abundant one.^9^ The membrane-anchored hCA IV and the transmembrane isoform hCA XIV are also expressed in many regions of the CNS, with hCA IV having the same expression pattern as hCA VII.^28^^,^^29^ Differently, the transmembrane proteins hCA IX and XII are hypoxia-induced enzymes that have been found overexpressed in numerous brain cancers such as glioblastoma multiforme.^30–32^ Finally, mitochondrial hCA VA and VB are present in the CNS, with VA being originally identified in neurons and astrocytes.^33–35^

**Figure 1.**
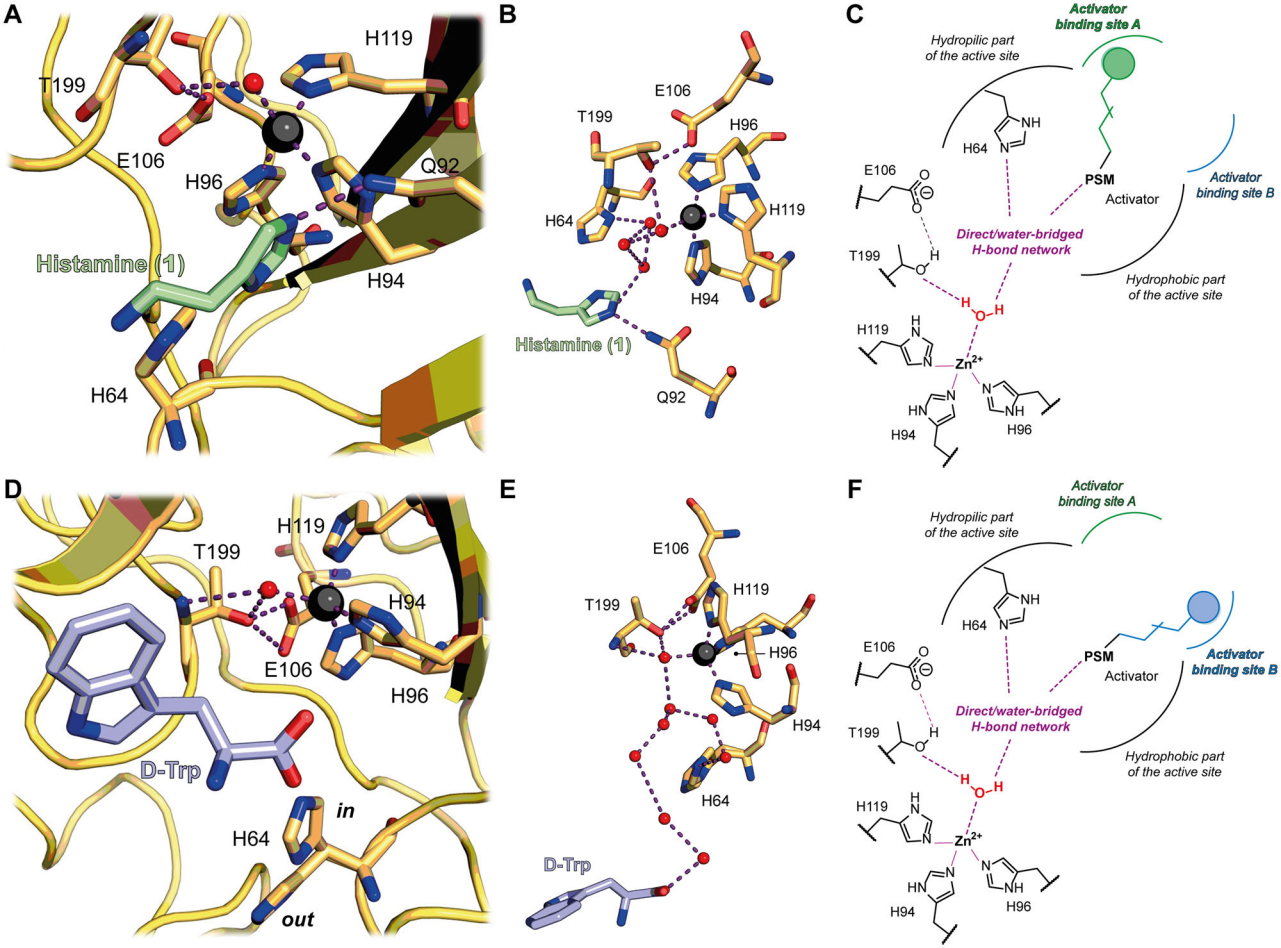
(A) Co-crystal structure of hCA II (yellow) in complex with histamine (**1**, shown in green) (PDB 1AVN).^36^ (B) Focus on the hydrogen bond network linking the Zn^2+^-bound water molecule to **1** (PDB 1AVN). (C) Schematic of CA activation mechanism for histamine-like compounds binding to the activator binding site A. Histamine (or analogous CAAs) bind to the activator binding site A and, through their PSM, facilitate proton transfer from Zn^2+^-bound H_2_O to the external medium. (D) Co-crystal structure of hCA II (yellow) in complex with D-Trp (shown in light blue) bound to (PDB 3EFI).^40^ (E) Focus on the hydrogen bond network linking the Zn^2+^-bound water molecule to D-Trp (PDB 3EFI). (F) Schematic of CA activation mechanism for D-Trp-like compounds binding to the activator binding site B. D-Trp (or analogous CAAs) bind to the activator binding site B and, through their PSM, facilitate proton transfer from Zn^2+^-bound H_2_O to the medium. In panels (A), (B), (D), and (E) the Zn^2+^ atom is shown as a black sphere, water molecules are displayed as red spheres, and polar interactions are depicted in purple.

Ever since the resolution of first X-ray crystal structure of a CA bound to a CA activator (CAA),^36^ which revealed their mode of action, the interest in CAAs has been constantly increasing. To date, the identified CAAs comprise amino acids, oligopeptides, biogenic amines such as histamine, serotonin, and catecholamines, and their derivatives.^5^ The first CA-CAA cocrystal structure showed the binding mode of histamine (**1**) to hCA II (Figure 1(A)) and indicated that CAAs bind at the entrance of the CA catalytic site.^36^ Here, CAAs facilitate the rate-limiting step of the catalytic cycle consisting of the proton transfer process from the Zn^2+^-coordinated water molecule to the external reaction medium (Figures 1(B,C)). In the apo protein, the residue responsible for proton transfer is histidine (His64 in hCA I, II and many other α-CAs), whose pK_a_ is ∼7, thereby being able to shuttle protons in a pH range of 6–8.^37^ Thus, following the formation of the enzyme-substrate-activator ternary complex as a consequence of CAA binding into a region close to His64, the proton transfer process to the medium becomes intramolecular, thus being more efficient than the intermolecular transfer from the enzyme to the solvent which is not stably bound inside the enzyme cleft [see Equation (2)].

(2) $${\mathrm{EZn}}^{2+}–{\mathrm{OH}}_{2}\;+\;A\;⇌[{\mathrm{EZn}}^{2+}–{\mathrm{OH}}_{2}–A]⇌[{\mathrm{EZn}}^{2+}–{\mathrm{OH}}^{–}–{\mathrm{AH}}^{+}]⇌\;{\mathrm{EZn}}^{2+}–{\mathrm{OH}}^{–}+{\mathrm{AH}}^{+}$$

Ever since **1** has been co-crystallised with hCA II, many other CAAs have been identified, and different co-crystal structures of hCA I/II in complex with CAAs have been reported, including those with L- and D-His,^38^ L- and D-Phe,^39^ D-Trp (Figures 1(D,E)),^40^ L-adrenaline,^41^ and pyridinium-based analogues of histamine.^42^ All CAAs possess proton shuttling moieties (PSMs) that are capable of binding and transferring protons from the enzyme to the external medium. They all bind to the so-called activator binding site A (Figure 1(C)), except for D-Trp, which binds to another region (activator binding site B), although its PSMs (both the amino and carboxylic acid portions) are located close to the activator binding site A (Figures 1(D–F)).^5^^,^^40^

From a therapeutic point of view, the primary applications of CAAs are in the fields of pharmacological augmentation of synaptic efficiency, spatial learning, and memory.^43^ Indeed, hCA activity was shown to be decreased in Alzheimer’s disease (AD) patients.^44^ Moreover, hCA activation was shown to increase memory performance by enhancing the ERK pathways, whose activation in the cortex and hippocampus is known to cause structural synaptic modifications that promote memory storage.^45–47^ Hence, CAAs may find therapeutic application in the treatment of post-traumatic stress disorders (PTSD), phobias, anxiety, and memory-related disorders linked to ageing and neurodegenerative diseases.^48^

In line with the importance of hCA activation in the CNS, many psychoactive drugs and therapeutically used histamine receptor modulators, including antihistamines, all of which bear potential PSMs (i.e. protonable moieties such as imidazole, amine, guanidine or its bioisosteres), have been recently indicated to also act as CAAs.^49^^,^^50^ Similarly, selective serotonin reuptake inhibitors antidepressants such as fluoxetine, sertraline, and citalopram have been shown to act as hCAI and II activators.^51^ In contrast, no antipsychotic drugs have been shown to activate CAs, with the atypical antipsychotics sulpiride and aripiprazole actually inhibiting hCAI/II and hCAI/II/IV, respectively.^52^^,^^53^ Overall, these data suggest that the therapeutic and/or adverse effects of many marketed drugs may be linked to a polypharmacology-based mode of action.

**Figure 2.**
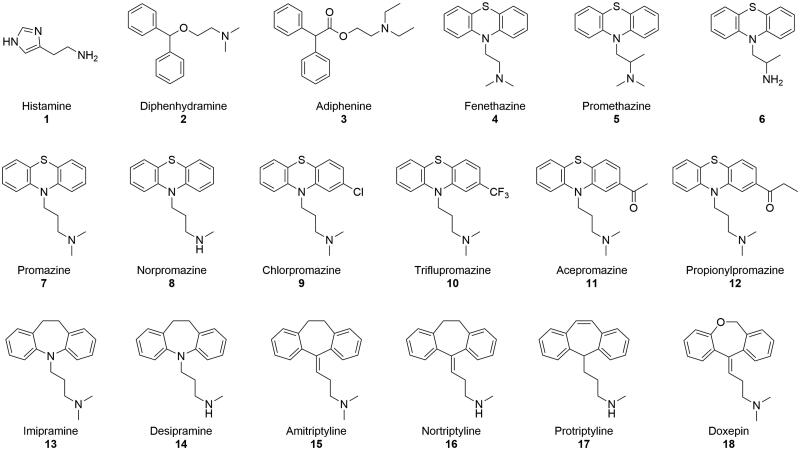
Chemical structures of histamine (**1**) and compounds **2**–**18** evaluated in this study as CAAs.

Nonetheless, there are still many drugs acting on the CNS and possessing protonable moieties that have not been evaluated yet for their hCA activation potential. These include the H_1_ receptor (H_1_R) inverse agonists diphenhydramine (**2**),^54^ the nicotinic receptor antagonist adiphenine (**3**),^55^ the phenothiazine-based H_1_R inverse agonists fenethazine (**4**) and promethazine (**5**),^54^ its bis desmethyl analogue **6**, the phenothiazine-based neuroleptic drugs promazine (**7**), **9–12**,^56^ the promazine desmethyl metabolite **8**,^57^^,^^58^ and the tricyclic antidepressants (TCA) **13–18** (Figure 2).^59^

Hence, through this study, we investigated the potential of such derivatives as activators of four pharmacologically significant CA isoforms: the cytosolic hCA I, II, and VII, and the membrane-anchored hCA IV.

## Materials and methods

### Chemistry

Compounds **1–3**, **5**, **7**, and **9–18** were commercially available, highest purity reagents from Sigma-Aldrich (Milan, Italy). Compounds **4**, **6**, and **8** were commercially available, highest purity derivatives from Ambinter SARL, Hong Kong Chemhere Co. Ltd, and Enamine Ltd, respectively.

### Carbonic anhydrase activation

The CA-catalysed CO_2_ hydration was monitored *via* a stopped-flow method^60^ using Phenol red as indicator and executing the measurements at the maximum absorbance of 557 nm, monitoring the initial rates of the CA-catalysed CO_2_ hydration reaction for 10 to 100 s. For every compound, at least six traces of the initial 5–10% of the reaction were employed for establishing the initial velocity. The uncatalysed rates were measured with the same approach and subtracted from the total rates. Stock solutions of each compound (0.1 mM) were prepared in distilled-deionised water and dilutions up to 0.1 nM were prepared using the assay buffer. The activation constant (*K*_A_), defined in a similar way as the inhibition constant (*K*_I_), was calculated using the classical Michaelis–Menten [Equation (3)], which was fitted using nonlinear least squares employing GraphPad Prism 3:

(3) $$v=\frac{v_{\max}}{1+\frac{K_{M}}{\left[S\right]}(1+\;\frac{{\left[A\right]}_{f}}{K_{A}})}\;$$

where [A]_f_ is the free concentration of the potential activator.

Since the assays were carried out at substrate concentrations significantly lower than the *K*_M_ ([S] ≪ *K*_M_), and because [A]_f_ can be defined in the form of the total concentration of the enzyme ([E]_t_) and activator ([A]_t_), the obtained competitive steady-state equation for calculating the activation constant is given by Equation (4):

(4) $$v=\frac{v_{0}K_{A}}{K_{A}+{\left[A\right]}_{t}-0.5{\left[({\left[A\right]}_{t}+{\left[E\right]}_{t}+K_{A})-{{\left([A\right]}_{t}+{\left[E\right]}_{t}+K_{A})}^{2}-4{\left[A\right]}_{t}{\left[E\right]}_{t}\right]}^{1/2}}$$

where *v_0_* represents the initial velocity of the enzyme-catalysed reaction in the absence of activator. All CA isoforms used in the experiments were purified recombinant proteins obtained as already reported.^61–65^ Enzyme concentrations in each assay were in the range of 7.0 − 12.0 nM.

## Results and discussion

As previously mentioned, histamine (**1**) was among the first uncovered CAAs, and histamine receptor modulators have been recently investigated for their potential as CAAs.^59^ Hence, we set out to assess the influence on hCA activity of yet unexplored antihistamines, such as the benzhydryloxyethylamine derivative **2** and phenothiazine derivatives **4** and **5**. We also evaluated the anticholinergic compound **3**, possessing a scaffold similar to **2**, but with an additional carbonyl spacer between the oxyethylamine and benzhydryl moieties, and compound **6**, the bis desmethyl derivative of **5**. Given the phenothiazine-based structure of the first-generation H1 antihistamines **4** and **5**, we went on to evaluate the CA activation potential of the phenothiazine-based neuroleptics **7**, and **9**–**12**, as well as the desmethyl metabolite of **7**, compound **8**.^57^^,^^58^ Finally, we also analysed TCAs **13**–**18**, structurally characterised by an expansion of the central ring of phenothiazines from a 6-membered to a 7-membered cycle (Figure 2). All the assayed compounds possess groups that may in principle act as PSM in the pH range 6–8, which represents an essential feature for CAAs.^37^^,^^66^

Based on the results obtained from the stopped-flow CO_2_ hydrase assay on the four hCA isoforms I, II, IV and VII (Table 1), the following observations may be drawn:

**Table 1.** hCA I, II, IV and VII activation with compounds **2–18** by a stopped-flow CO_2_ hydrase assay. Histamine (**1**) used as reference CAA.

Compd	**K_A_ (µM)** ^a^				Literature Data	
	hCA I	hCA II	hCA IV	hCA VII	CNS action	Reference(s)
**1**	2.1	125	25.3	37.5	+	^67^
**2**	>50	>50	>50	4.7	+	^68^ ^,^ ^69^
**3**	10.2	28.6	30.2	3.4	+	^70^ ^,^ ^71^
**4**	>50	13.7	45.0	9.1	+	^72^
**5**	>50	7.0	>50	29.5	+	^69^ ^,^ ^73^
**6**	>50	4.8	>50	9.8		
**7**	>50	3.7	>50	2.5	+	^74^ ^,^ ^75^
**8**	>50	1.4	27.6	8.9		
**9**	>50	2.8	43.9	5.2	+	^75^ ^,^ ^76^
**10**	23.6	1.9	>50	4.8	+	^75^
**11**	>50	21.2	>50	3.0	+	^77^
**12**	>50	11.3	28.5	2.5	+	^78^
**13**	9.3	2.0	27.8	3.1	+	^79^
**14**	24.8	4.5	10.0	1.6	+	^80^
**15**	>50	18.9	>50	13.3	+	^81^ ^,^ ^82^
**16**	>50	11.7	>50	6.2	+	^82^
**17**	>50	42.1	>50	4.9	+	^83^
**18**	>50	45.6	>50	9.2	+	^84^

^a^ Results of three different assays (*n* = 3; errors within ± 10% of the reported values). **+** means that there is evidence of BBB crossing and central action; **no sign** means that no literature data are available.

- Only four compounds exhibited cytosolic hCA I activation (K_A_ < 50 µM) and none of them outperformed **1** in increasing hCA I activity, with the most potent ones being **3** (K_A_ = 10.2 µM) and **13** (K_A_ = 9.3 µM). The demethylation of **13** decreased hCA I activation potency, as demonstrated by the K_A_ value measured for the mono desmethyl analogue **14**, which is >2.5-fold higher (K_A_ = 24.8 µM).
- The cytosolic hCA II was activated by all tested compounds, apart from **2**. Specifically, all compounds exhibited improved K_A_ values over **1** for this isoform. Phenothiazines **6–10**, and TCAs **13** and **14** displayed K_A_ values lower than 5 µM, with norpromazine (**8**) being the most potent hCA II activator (K_A_ = 1.4 µM), immediately followed by triflupromazine (**10**) and imipramine (**13**), with K_A_ values of 1.9 and 2.0 µM, respectively. Notably, demethylation had divergent effects on hCA II activation for **13** and the rest of the assayed compounds. Indeed, the double demethylation of **5** and the mono demethylation of **7**, leading to **6** and **8**, respectively, increased the hCA II activation profile by 1.5- to 2.6-fold. Similarly, removal of a methyl group from **15** augmented hCA II activation [K_A_(**15**) = 18.9 µM, K_A_(**16**) = 11.7 µM], while the opposite effect was observed in the case of **13**, since its mono desmethyl derivative **14** was ∼2-fold less potent.
- The membrane-anchored hCA IV displayed an activation profile analogous to that observed for hCA I, with only few molecules capable of enhancing its activity. The only compound exhibiting a hCA IV activation more efficient than **1** was **14**, with a K_A_ value of 10.0 µM. Notably, in this case, methylation of the exocyclic nitrogen decreased CA activation potency, with **13** showing a ∼2.8-fold higher K_A_. The same pattern could be observed for phenothiazines **7** and **8**, with the mono desmethyl derivative **8** having a K_A_ value of 27.6 µM, while **7** was essentially not active (K_A_ > 50 µM).
- The cytosolic hCA VII was efficiently activated by the compounds investigated in the study, and all of them possessed an improved activation profile compared to **1**, with a wide range of derivatives being active in the low micromolar range. Specifically, half of the assayed compounds exhibited a K_A_ value lower than 5 µM. These include: (i) the benzhydryl derivatives **2** and **3**; (ii) the phenothiazine-based compounds **7**, **10**, **11**, and **12**; (iii) the TCAs **13**, **14**, and **17**. Among the tested molecules, the most promising hCA VII activator was desipramine **14** (K_A_ = 1.6 µM), the mono desmethyl analogue of **13**, which could still activate hCA VII, albeit almost 2-fold less efficiently. The same structure-activity relationship (SAR) was observed for TCAs **15** and **16**, with the mono desmethyl derivative being >2-fold more potent than its parent compound **15.** Conversely, the phenothiazines bearing a 1,3-diaminopropane chain (**7** and **8**) followed the opposite trend, with **7** being ∼3.5-fold more potent than the mono desmethyl analogue **8**. In the case of the phenothiazine-based 1,2-diaminopropane compounds **5** and **6**, removal of both *N*-methyl groups led to a 3-fold decrease in CA VII activation potency. Interestingly, compound **2**, which was inactive towards all other isoforms, was a good hCA VII activator (K_A_ = 4.7 µM). Similarly, TCAs **17** and **18**, which were only weakly potent towards hCA II, could both activate hCA VII with K_A_ values of 4.9 and 9.2 µM, respectively.

By looking at the different chemical classes of the analysed compounds, we could also draw some interesting SAR:

- The benzhydryl compounds **2** and **3** preferentially activated hCA VII, with **2** being hCA VII-selective, while **3** was one of the few pan-hCA activators among the assayed compounds.
- Both phenothiazine-based antihistamines (**4**, **5**, and the bis desmethyl derivative **6**) and neuroleptics (**7**–**12**) displayed selective hCA II/VII activation properties. Specifically, compound **7** and its derivatives **9** and **10**, possessing an electron-withdrawing moiety at C2 of the phenothiazine core, displayed K_A_ values in the low micromolar range for both hCA II and VII. In this series, compounds **4** and **5** and, partly, **6** were slightly less potent towards both isoforms, and this may be ascribed to the shorter 2-carbon spacer between the endocyclic and exocyclic nitrogen atoms. Moreover, **11** and **12** suffered from a significant drop in hCA II activation compared to analogues **7**–**10**. In this case, the only difference between the tested compounds is the presence of a bulkier substituent at C2 of the phenothiazine core in **11** and **12**.
- TCAs were endowed with a similar activation pattern to phenothiazines, with all compounds preferentially activating hCA II and VII. In this case, while compounds **15**-**18** were selective for hCA II/VII, **13** and its mono desmethyl derivative **14** displayed a non-selective activation profile, with K_A_ values of 10 µM or less towards hCA I and hCA IV, respectively. Although being less selective, **13** and **14** were the most potent activators of hCA II and VII in this series. Indeed, modification of some key structural features led to a drop in potency, particularly emphasised in the case of hCA II. According to the data, replacement of the endocyclic electron-rich nitrogen with a sp^2^ carbon and the consequent generation of a 5-exocyclic double bond (compounds **15**, **16**, **18**) was detrimental for compound activity. Moreover, in compounds **17** and **18**, the ethylene bridge between the two phenyl rings was replaced by a double bond or by inserting an oxygen atom, and this further modification especially affected their hCA II activation potency. As a result, the 10,11-dihydro-5*H*-dibenzo[*b,f*]azepine core represented the preferred chemotype for hCA II/VII activation, despite its lack of selectivity. On the other hand, the 5*H*-dibenzo[*a,d*][7]annulene core offered a fair compromise between hCA activation and selectivity, as exemplified by compound **17**, the most hCA VII-selective activator among the tested TCAs.

Finally, as illustrated in the "Literature Data" section of Table 1, most of the examined compounds have been shown to cross the blood-brain barrier (BBB) and have CNS effects. As a result, hCA activation in the CNS may contribute to their pharmacological activity.

## Conclusions

In this study, we examined the hCA activating properties of a variety of clinically-approved drugs (or derivatives thereof, Figure 2) known to act at CNS level such as antihistamines, neuroleptics, or antidepressants. The compounds have been evaluated on four hCA isoforms expressed in the CNS, namely hCA I, II, IV, and VII. All the assessed compounds displayed activation of at least one hCA isoform, which is consistent with the fact that they all include portions that may act as PSM at pH values ranging from 6 to 8. The compound series displayed lower activation of hCA I and IV while being generally more efficient in activating hCA II and VII. Specifically, the benzhydryl amines **2** and **3** displayed preferential activation towards hCA VII, while both phenothiazine-based compounds and TCAs could activate both hCA II and VII, with an overall slight preference of phenothiazines towards hCA II. Among the tested compounds, the phenothiazine derivative promazine (**7**) displayed the best profile in terms of potency and hCA II/VII selectivity over the other hCA isoforms. TCAs **13** and **14**, on the other hand, were essentially non-selective, and modifications to their 5*H*-dibenzo[*b,f*]azepine core, although improving the selectivity towards hCA VII, lowered compound potency, as evidenced by the activity profiles of **17** and **18**.

Remarkably, hCA VII was overall the most efficiently activated isoform by the compounds evaluated in our analysis. This is of significant relevance since hCA VII is the most abundant CA in the human CNS^9^ and is involved in the maintenance of the bicarbonate gradient in neurons as well as neuronal signalling.^7^ It is therefore of great interest that all tested compounds, belonging to a diverse subset of chemical and pharmacological classes, were identified as activators of hCA VII. These molecules may serve as lead compounds for drug discovery campaigns aiming at developing novel CNS-specific CAAs.

Furthermore, the evidence collected through this study offers additional insights into the pharmacological profile and therapeutic effects of clinically employed drugs such as antihistamines, phenothiazine-based antipsychotics, and TCAs. Indeed, both their therapeutic activity and adverse effects are connected to polypharmacology-based mechanisms, and the enhancement of hCA activity should now be considered. Ultimately, the present research may provide novel perspectives into the complex network linking hCA function and CNS homeostasis. Indeed, while CAAs have only recently been considered for their pharmacological applications in memory and cognition therapy,^43^^,^^45^^,^^47^^,^^48^ their potential is undeniable, and research aimed at investigating hCA activation at central level is critical for the development of new drugs with possible applications in neurodegeneration.
